# Minimally Invasive Percutaneous Plate Osteosynthesis (MIPPO) Versus Intramedullary Interlocking Nailing (IMILN) in Extra-articular Proximal Third Tibial Fractures: A Prospective Comparative Study

**DOI:** 10.7759/cureus.111947

**Published:** 2026-07-02

**Authors:** Sushit K Roul, Aditya Acharya, Tushar A Rath, Rupanjan Sarma, Tarun Garg, Shresth Kumar

**Affiliations:** 1 Orthopaedics, Institute of Medical Sciences and Sum Hospital, Bhubaneswar, IND

**Keywords:** ao 41a2, extra-articular fracture, intramedullary nailing, locking plate, mippo, poller screw, proximal tibia fracture, tibial metaphyseal fracture

## Abstract

Background and objective

Extra-articular fractures of the proximal third of the tibia are technically demanding injuries due to the complex deforming muscular forces and frequently compromised soft tissue envelope. The optimal treatment strategy for this fracture subtype remains unsettled, particularly regarding the choice between minimally invasive percutaneous plate osteosynthesis (MIPPO) and intramedullary interlocking nailing (IMILN). This study was undertaken to compare these two treatment modalities and determine their relative effectiveness.

Methods

A single-center, prospective, comparative study employing alternating allocation was conducted at IMS and SUM Hospital, Bhubaneswar, with patient recruitment carried out from September 2024 to February 2025, and final clinical and functional follow-up at 12 months completed by March 2026. Fifty-two adults aged 20-60 years with AO/OTA 41A2 fractures, including Gustilo-Anderson grade I and II open injuries, were sequentially allocated to either MIPPO (n = 26) or IMILN (n = 26). The primary outcomes included time to radiological union and 12-month functional assessment using the Knee Society Score (KSS), Lower Extremity Functional Scale (LEFS), and Johner and Wruhs grading scale. Continuous variables were compared using the independent-samples t-test, while categorical variables were analyzed using the chi-square or Fisher’s exact test as appropriate. Statistical analyses were performed using IBM SPSS Statistics version 21.0 (IBM Corp., Armonk, NY).

Results

Forty-six patients completed the one-year follow-up (MIPPO, n = 24; IMILN, n = 22). Mean radiological union time was 16.1 weeks (MIPPO) versus 16.9 weeks (IMILN) (mean difference: −0.8 weeks, 95% CI: −4.0 to 2.4; p = 0.62). Final union rates were 23/24 (95.8%) and 21/22 (95.5%), respectively. Malreduction > 5° occurred in 4/26 (15.4%, MIPPO) and 5/26 (19.2%, IMILN) (p = 0.71). Mean KSS was 80.2 versus 80.8 (mean difference: −0.6, 95% CI: −7.0 to 5.8; p = 0.85), and mean LEFS was 65.2 versus 67.3 (mean difference: −2.1, 95% CI: −6.6 to 2.4; p = 0.35). Excellent or good Johner and Wruhs grades were achieved in 18/24 (75.0%, MIPPO) and 18/22 (81.8%, IMILN). Partial weight-bearing was initiated earlier in the IMILN group (two to three postoperative days vs. three to four weeks; p < 0.001). Intraoperative adjuncts were required in 13/26 (50.0%) of IMILN versus 3/26 (11.5%) of MIPPO procedures (p = 0.005).

Conclusions

Within the limitations of this small, single-center cohort study, MIPPO and IMILN demonstrated comparable union rates and one-year functional outcomes for AO/OTA 41A2 fractures. IMILN facilitated earlier mobilization but necessitated adjunct procedures more frequently and was associated with anterior knee pain. MIPPO was associated with minimal knee-related morbidity, albeit with a more gradual rehabilitation timeline. Implant selection should therefore be guided by fracture morphology, soft-tissue condition, and locally available instrumentation rather than an absolute preference for either construct.

## Introduction

Extra-articular fractures of the proximal third of the tibia constitute approximately 5-11% of tibial shaft fractures [1]. The combination of a flared metaphysis, cancellous bone, and the deforming pull of the patellar tendon on a short proximal fragment makes these injuries technically demanding to fix and to maintain reduction [2]. High-energy mechanisms predominate, and a substantial proportion are open or have compromised overlying soft tissues [1,2].

Two minimally invasive options now predominate in the operative management of these injuries. Locking-plate fixation introduced through a submuscular tunnel, or minimally invasive percutaneous plate osteosynthesis (MIPPO), provides angular stability that is largely independent of bone quality and minimizes the soft-tissue morbidity associated with conventional open plating [3]. Closed intramedullary interlocking nailing (IMILN) is a load-sharing construct that facilitates earlier rehabilitation and avoids dissection over the anterolateral aspect of the leg [2]. Each technique has well-described failure modes. Plate fixation carries a risk of implant-related soft-tissue irritation and, in open injuries, deep infection [4]. Standard infrapatellar nailing of the proximal tibia has historically been associated with apex-anterior and valgus malalignment, particularly when the entry point is too medial or when the proximal fragment is short [4,5].

Several technical refinements have narrowed this gap. A proximal-lateral entry point, Poller (blocking) screws [6,7], temporary unicortical plating, and the femoral distractor each address different components of the deforming forces [4,6,7,8]. Suprapatellar (semi-extended) nailing, first described by Tornetta and Collins [5] and increasingly adopted over the last decade, reduces patellar tendon tension on the proximal fragment during nail insertion [5]. Recent comparative work and meta-analyses suggest a measurable improvement in coronal and sagittal alignment when the suprapatellar route is used, alongside lower rates of anterior knee pain [8-17].

Despite the large body of single-arm evidence, head-to-head comparative trials limited to a homogeneous AO 41A2 cohort remain relatively scarce. The most recent prospective comparative cohort study (Teimouri et al., 2023 [13]) and the 2017 systematic review by Liu and Cen [18] both concluded that union and complication profiles were broadly similar between plating and nailing techniques, although they noted substantial heterogeneity in fracture patterns and surgical techniques across the included studies. We also ensured standardized reporting of open injuries using the Gustilo-Anderson classification [19].

The primary objective of this study was to compare radiological union timelines and 12-month functional outcomes (using the Knee Society Score (KSS), Lower Extremity Functional Scale (LEFS), and Johner and Wruhs grading) between MIPPO and IMILN in AO/OTA 41A2 extra-articular proximal tibial fractures [20,21,22]. The secondary objectives were to compare postoperative axial alignment maintenance, overall complication profiles, specific postoperative rehabilitation weight-bearing timelines, and the operational requirements for intraoperative adjunctive reduction techniques. Our working hypothesis was that the two constructs would yield comparable functional outcomes at one year, with any differences largely limited to rehabilitation timelines and the need for intraoperative adjuncts to achieve and maintain reduction.

## Materials and methods

Study design and setting

A prospective, single-center comparative cohort study employing an alternating allocation strategy was conducted at the Department of Orthopaedic Surgery, IMS and SUM Hospital, Bhubaneswar, Odisha, India. Patient enrolment was conducted between September 2024 and February 2025, followed by an active postoperative follow-up period through March 2026, ensuring a minimum follow-up duration of 12 months for all participants. To reduce operator variability and minimize technical confounding, all procedures in both study groups were performed by the same surgical team, comprising two experienced senior orthopedic surgeons, in accordance with standardized operative protocols.

The study protocol received approval from the Institutional Ethics Committee (IEC No. IEC/IMS.SH/SOA/2024/810A). The investigation was conducted in accordance with the principles of the Declaration of Helsinki (2013 revision). Written informed consent was obtained from all participants before enrolment. Because treatment allocation was not concealed and neither the operating surgeons nor the outcome assessors were blinded, the study does not qualify as a randomized controlled trial. Accordingly, it is presented as a prospective comparative study using an alternating allocation method.

Sample size

No formal a priori sample size calculation was performed. This study represents a prospective institutional cohort accrued over the study period and may be underpowered to detect small differences in infrequent outcomes.

Participants

Consecutive patients with proximal tibial fractures presenting during the study period were screened. The inclusion criteria were as follows: age 20-60 years; AO/OTA 41A2 extra-articular metaphyseal wedge fracture of the proximal third; closed fracture or Gustilo-Anderson grade I or II open fracture [19]; willingness to comply with follow-up. The exclusion criteria were as follows: Gustilo-Anderson grade III open injury; AO 41A3 highly comminuted metaphyseal fracture; intra-articular extension (41B/C); established compartment syndrome; pre-existing ipsilateral deformity or previous surgery on the affected limb; pathological fracture; distal neurovascular deficit; inability to consent.

Allocation

Eligible patients were allocated to the MIPPO or IMILN groups in a strict alternating sequence on the day of admission (odd-numbered arrivals to MIPPO; even-numbered to IMILN). There was no allocation concealment and no stratification by fracture subtype. The potential for selection bias associated with this allocation method is acknowledged in the Limitations section.

Preoperative assessment

Anteroposterior and lateral radiographs of the leg, knee, and ankle were obtained at presentation. Fractures were classified per AO/OTA by two consultant orthopedic surgeons [1]. Open fractures were graded by the Gustilo-Anderson classification [19] at initial wound debridement and received intravenous first-generation cephalosporins. A first-generation cephalosporin was given 30 minutes before incision in all cases and continued for five postoperative days in open injuries.

Surgical technique - MIPPO

Procedures were performed under spinal anesthesia with the patient supine on a radiolucent table. Manual traction under image-intensifier control was used for indirect reduction. A 4-5 cm anterolateral incision was made proximal to the fracture, and a submuscular tunnel was developed across the fracture zone with a periosteal elevator. Standardized hardware consisting of 4.5 mm titanium alloy locking compression plates (LCP), hockey stick locking plates, or buttress plates was advanced submuscularly, provisionally held with Kirschner wires (K-wires) and pointed reduction clamps, and fixed distally through stab incisions under fluoroscopy. Proximal screws (locking and cancellous) were placed through a small proximal incision. Postoperatively, a temporary long-leg posterior slab was maintained for two weeks. This temporary immobilization protocol was utilized strictly to facilitate uncompromised soft-tissue healing, reduce acute post-traumatic edema, and protect the wounds across both closed and open sub-cohorts until the definitive stitch removal period at 12-15 days, immediately after which aggressive active mobilization was commenced.

Surgical technique - IMILN

With the patient supine and the knee flexed over a radiolucent bolster, a patellar tendon-splitting approach was used in all cases. The entry point was positioned proximally and laterally, in line with the lateral intercondylar eminence, a modification reported to reduce valgus and apex-anterior malalignment [4,8]. Awl entry was performed in maximum tolerated flexion; the knee was then extended somewhat to allow guide-wire passage. Reduction was confirmed in both planes before sequential reaming. An interlocking tibial nail was introduced over the guide wire utilizing standard multi-axial titanium nails ranging from 8 mm, 9 mm, and 10 mm in diameter, with length and structural diameter selection determined via preoperative digital template planning and confirmed following sequential intraoperative manual reaming. Care was taken not to countersink more than 2-3 mm below the entry cortex. Proximal locking used the targeting jig; distal locking was free-hand under image control. Adjunctive measures (femoral distractor, Poller/blocking screws, or temporary anterior unicortical plating) were used at the surgeon’s discretion when standard technique did not yield satisfactory reduction [6,7].

Postoperative rehabilitation

Active knee and ankle exercises with isometric quadriceps strengthening were started on postoperative days two to three in both groups. Sutures were removed on days 12-15. IMILN patients began toe-touch partial weight-bearing on days two to three. MIPPO patients remained non-weight-bearing for three to four weeks, after which graded partial weight-bearing was advanced according to construct stability. Full weight-bearing was authorized in both groups once bridging callus was visible on three of four cortices on plain radiographs.

Follow-up and outcome measures

Patients were reviewed at two, six, 10, 14, 18, and 24 weeks, then at nine and 12 months. Radiological union was defined as bridging callus on three of four cortices with loss of the fracture line. Acceptable alignment was < 5° angulation in any plane and < 10° rotational discrepancy. Delayed union was defined as no union by 24 weeks; non-union as absence of progressive callus beyond 24 weeks despite adequate fixation. Twelve-month outcome measures were the KSS (KSS; 0-100; excellent ≥ 85, good 70-84, fair 60-69, poor < 60) [20], LEFS (LEFS; maximum 80) [21], and the Johner and Wruhs criteria [22]. Outcome assessment and radiographic reviews were performed directly by the two senior operating surgeons from the primary treatment team; independent blinded review was not feasible. This internal assessment process may have introduced observer bias, and this potential limitation is explicitly acknowledged in the Limitations section.

Statistical analysis

Data were analyzed using IBM SPSS Statistics v.21.0 (IBM Corp., Armonk, NY). Normality of continuous variables was assessed with the Shapiro-Wilk test. Continuous outcomes are summarized as the group means with their 95% confidence intervals (CI), together with the 95% CI of the between-group difference, the test statistic (t with degrees of freedom), and the exact p-value. Categorical variables are summarized as counts and percentages and compared with the chi-square or Fisher’s exact test, the latter labeled where used. Between-group comparisons of continuous data used the independent-samples t-test for normally distributed variables and the Mann-Whitney U test for non-normal data. Exact p-values are reported throughout; a p-value < 0.05 was considered statistically significant.

Participant flow

**Figure 1:**
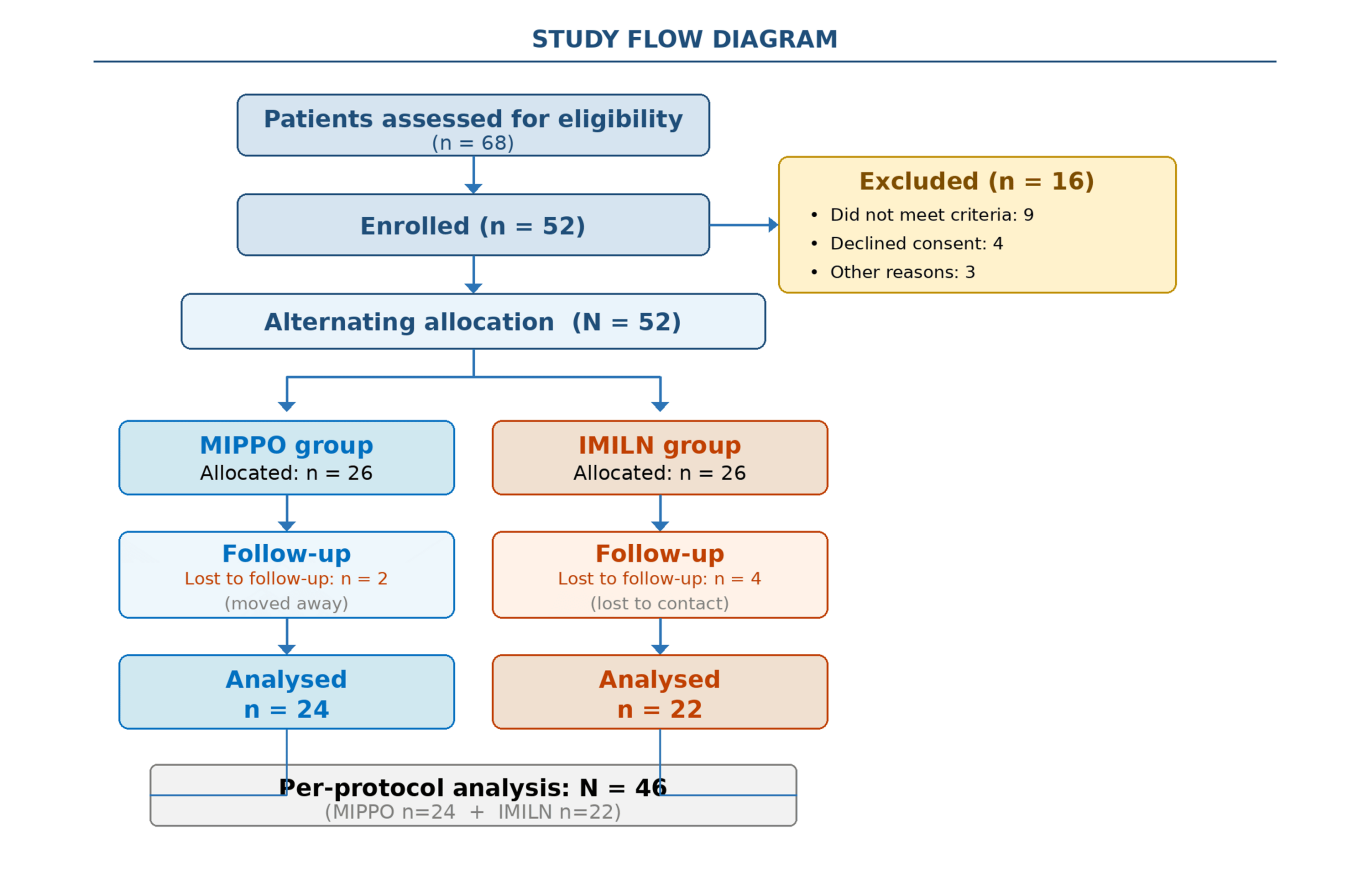
Study flow diagram Patient screening, alternating allocation to MIPPO (n = 26) and IMILN (n = 26), follow-up, loss to follow-up, and per-protocol analysis (MIPPO, n = 24; IMILN, n = 22) MIPPO: minimally invasive percutaneous plate osteosynthesis; IMILN: intramedullary interlocking nailing

Sixty-eight patients were screened; 52 met inclusion criteria, and 52 were enrolled (26 per arm). Six patients (MIPPO, n = 2; IMILN, n = 4) were lost to follow-up before 12 months; 46 patients (MIPPO, n = 24; IMILN, n = 22) completed assessment and formed the per-protocol cohort. The flow of participants is summarized in Figure 1.

## Results

Baseline characteristics

The cohort included 43 men (43/52, 82.7%) and nine women (9/52, 17.3%), with a mean age of 40.2 years (range: 20-60 years). The 31-40-year age group was the most commonly represented (20/52, 38.5%). Road traffic accidents (RTAs) constituted the predominant mechanism of injury (44/52, 84.6%), while the right limb was affected in 29/52 (55.8%) patients. Of the fractures, 30/52 (57.7%) were closed, 17/52 (32.7%) were classified as Gustilo-Anderson grade I open, and 5/52 (9.6%) as grade II open. Baseline patient demographics and preoperative injury characteristics were well balanced between the MIPPO and IMILN groups, with no statistically significant differences observed in any evaluated parameters, including active smoking status, controlled diabetes mellitus, or the presence of an associated ipsilateral fibula fracture.

BMI was not recorded, as accurate, standardized height and weight measurements were not possible during the acute post-traumatic period because patients remained immobilized. The two groups were comparable at baseline (Table 1). The mean injury-to-surgery interval was significantly shorter in the IMILN group (4.23 days, 95% CI: 3.5 to 4.9) than in the MIPPO group (5.46 days, 95% CI: 4.7 to 6.2), with a mean difference of 1.23 days (95% CI: 0.2 to 2.2; t(50) = 2.52, p = 0.015), likely reflecting the additional preoperative planning required for MIPPO. In contrast, the mean operative time was similar between groups (76.1 min, 95% CI: 71.5 to 80.7, vs. 81.9 min, 95% CI: 77.3 to 86.5; mean difference: −5.8 min, 95% CI: −12.1 to 0.5; t(50) = 1.85, p = 0.07.

**Table 1:** Baseline demographics and injury characteristics (N = 52) Continuous variables: independent-samples t-test, summarized as group means with the 95% CI of the difference where a comparison is drawn. Categorical variables: chi-square or Fisher’s exact test MIPPO: minimally invasive percutaneous plate osteosynthesis; IMILN: intramedullary interlocking nailing; CI: confidence interval; RTA: road traffic accident

Variable	MIPPO (n = 26)	IMILN (n = 26)	Test statistic (p-value)
Mean age, years (95% CI)	40.5 (36.5–44.5)	39.9 (35.9–43.9)	t(50) = 0.22 (p = 0.83)
Male, n (%)	22 (84.6)	21 (80.8)	χ²(1) = 0.13 (p = 0.71)
Right limb, n (%)	14 (53.8)	15 (57.7)	χ²(1) = 0.08 (p = 0.78)
RTA mechanism, n (%)	22 (84.6)	22 (84.6)	χ²(1) = 0.00 (p = 1.00)
Closed fracture, n (%)	16 (61.5)	14 (53.8)	χ²(1) = 0.32 (p = 0.58)
Gustilo–Anderson I, n (%)	8 (30.8)	9 (34.6)	χ²(1) = 0.09 (p = 0.77)
Gustilo–Anderson II, n (%)	2 (7.7)	3 (11.5)	Fisher’s exact (p = 0.64)
Controlled diabetes mellitus, n (%)	7 (26.9)	5 (19.2)	χ²(1) = 0.43 (p = 0.51)
Active smoking history, n (%)	8 (30.8)	7 (26.9)	χ²(1) = 0.09 (p = 0.76)
Associated ipsilateral fibula fracture, n (%)	11 (42.3)	12 (46.2)	χ²(1) = 0.08 (p = 0.78)
Mean injury-surgery interval, days (95% CI)	5.46 (4.7–6.2)	4.23 (3.5–4.9)	t(50) = 2.52 (95% CI 0.2 to 2.2; p = 0.015)
Mean operative time, min (95% CI)	76.1 (71.5–80.7)	81.9 (77.3–86.5)	t(50) = 1.85 (95% CI −12.1 to 0.5; p = 0.07)

Intraoperative adjuncts

Additional manoeuvres to obtain or maintain fracture alignment were required significantly more often in the IMILN group (13/26, 50.0%) than in the MIPPO group (3/26, 11.5%) (χ²(1) = 9.03, p = 0.005). Among patients in the IMILN group, a femoral distractor was used in five cases (5/26, 19.2%), Poller (blocking) screws in four (4/26, 15.4%), and temporary unicortical plating in four (4/26, 15.4%). In contrast, the MIPPO group required only a femoral distractor, which was used in three cases (3/26, 11.5%).

Radiological union and alignment

Primary union after the index procedure was achieved in 22/24 MIPPO cases (91.7%) and 19/22 IMILN cases (86.4%). Following secondary procedures (autologous bone grafting in two patients and dynamization in two patients), the final union rate increased to 23/24 (95.8%) in the MIPPO group and 21/22 (95.5%) in the IMILN group. The majority of fractures in both groups achieved union between 12 and 15 weeks (MIPPO, 62.5%; IMILN, 54.5%). The mean radiological union time was 16.1 weeks (95% CI: 13.8 to 18.4) for MIPPO and 16.9 weeks (95% CI: 14.5 to 19.3) for IMILN (mean difference: −0.8 weeks, 95% CI: −4.0 to 2.4; t(44) = 0.50, p = 0.62). The distribution of union times is presented in Table 2.

**Table 2:** Radiological union time distribution ^†^The four time bands were compared as a single distribution using Fisher’s exact test (p = 0.84), which yields an exact p-value without a separate test statistic; the band rows are descriptive. Mean union time: between-group difference: −0.8 weeks (95% CI: −4.0 to 2.4), independent-samples t-test MIPPO: minimally invasive percutaneous plate osteosynthesis; IMILN: intramedullary interlocking nailing; CI: confidence interval

Time to union	MIPPO, n (%) (n = 24)	IMILN, n (%) (n = 22)	Statistical test (p-value)
12–15 weeks	15 (62.5)	12 (54.5)	Fisher’s exact (p = 0.84)^†^
16–20 weeks	4 (16.7)	6 (27.3)	^†^
21–24 weeks	3 (12.5)	2 (9.1)	^†^
> 24 weeks	2 (8.3)	2 (9.1)	^†^
Mean union time, weeks (95% CI)	16.1 (13.8–18.4)	16.9 (14.5–19.3)	t(44) = 0.50 (p = 0.62)

Overall malreduction > 5° was observed in 4/26 MIPPO patients (15.4%) and 5/26 IMILN patients (19.2%) (Fisher’s exact test, p = 0.71) (Table 3). Apex-anterior angulation was the dominant deformity in both groups (MIPPO 2/26, 7.7%; IMILN 3/26, 11.5%). One patient in each group had varus malalignment; one IMILN patient had valgus, and one MIPPO patient had apex-posterior angulation. No patient had rotational malalignment or shortening > 1.5 cm.

**Table 3:** Postoperative alignment patterns (allocated cohort: n = 26 per arm) ^‡^Acceptable versus malreduced (> 5°) alignment was compared as a single dichotomy using Fisher’s exact test (p = 0.71), which yields an exact p-value without a separate test statistic; the individual deformity types are descriptive sub-categories of this comparison. Malreduction defined as > 5° angulation in any plane on standing radiographs MIPPO: minimally invasive percutaneous plate osteosynthesis; IMILN: intramedullary interlocking nailing

Alignment status	MIPPO, n (%) (n = 26)	IMILN, n (%) (n = 26)	Statistical test (p-value)
Acceptable (< 5°)	22 (84.6)	21 (80.8)	Fisher’s exact (p = 0.71)^‡^
Apex anterior	2 (7.7)	3 (11.5)	^‡^
Varus	1 (3.8)	1 (3.8)	^‡^
Valgus	0 (0)	1 (3.8)	^‡^
Apex posterior	1 (3.8)	0 (0)	^‡^
Total malreduction (> 5°)	4 (15.4)	5 (19.2)	Fisher’s exact (p = 0.71)

Complications

Superficial wound infection occurred in 4/24 MIPPO patients (16.7%) and 3/22 IMILN patients (13.6%); all cases resolved with intravenous antibiotics and wound care. One MIPPO patient (4.2%) developed a deep infection requiring operative débridement and local antibiotic bead application, with fracture union ultimately achieved at 25 weeks; no deep infections were observed in the IMILN group. Implant-related soft-tissue irritation was noted in two MIPPO patients (8.3%); however, neither required premature hardware removal during the follow-up period. Anterior knee pain was reported by two IMILN patients (2/22, 9.1%). One patient in the MIPPO group experienced common peroneal nerve (CPN) neurapraxia secondary to surgical retraction, which resolved spontaneously. No fixation failure, intraoperative fracture propagation, or postoperative compartment syndrome was observed. Table 4 summarizes the complication profile.

**Table 4:** Complication profile ^*^Malreduction denominator is the allocated cohort (n = 26 per arm); other complications use evaluable cohort denominators. Each complication is an independent yes/no outcome; Fisher’s exact test was used throughout because expected cell counts were < 5, and Fisher’s exact test reports an exact p-value without a test statistic MIPPO: minimally invasive percutaneous plate osteosynthesis; IMILN: intramedullary interlocking nailing; CPN: common peroneal nerve; NE: not estimable (no events in either group)

Complication	MIPPO, n (%) (n = 24)	IMILN, n (%) (n = 22)	Test (p-value)
Superficial infection	4 (16.7)	3 (13.6)	Fisher’s (p = 1.00)
Deep infection	1 (4.2)	0 (0)	Fisher’s (p = 1.00)
Non-union	1 (4.2)	1 (4.5)	Fisher’s (p = 1.00)
Delayed union	1 (4.2)	2 (9.1)	Fisher’s (p = 0.60)
Malreduction (> 5°)^*^	4/26 (15.4)	5/26 (19.2)	Fisher’s (p = 0.71)
Implant irritation	2 (8.3)	0 (0)	Fisher’s (p = 0.49)
Anterior knee pain	0 (0)	2 (9.1)	Fisher’s (p = 0.22)
CPN neurapraxia	1 (4.2)	0 (0)	Fisher’s (p = 1.00)
Compartment syndrome	0 (0)	0 (0)	NE
Fixation failure	0 (0)	0 (0)	NE

Functional outcomes

No statistically significant between-group differences in functional outcome were observed at 12 months. Mean KSS was 80.2 (95% CI: 75.7 to 84.7) for MIPPO and 80.8 (95% CI: 76.1 to 85.5) for IMILN (mean difference: −0.6, 95% CI −7.0 to 5.8; t(44) = 0.19, p = 0.85). Excellent or good KSS was recorded in 17/24 (70.8%) MIPPO and 17/22 (77.3%) IMILN patients. Mean LEFS was 65.2 (95% CI: 62.2 to 68.2) for MIPPO and 67.3 (95% CI: 64.1 to 70.5) for IMILN (mean difference −2.1, 95% CI: −6.6 to 2.4; t(44) = 0.94, p = 0.35). Excellent or good Johner and Wruhs results were obtained in 18/24 (75.0%) of MIPPO patients and 18/22 (81.8%) of IMILN patients (Table 5).

**Table 5:** Functional outcomes at 12 months Mean KSS: between-group difference −0.6 (95% CI: −7.0 to 5.8); t(44) = 0.19, p = 0.85. Mean LEFS: difference −2.1 (95% CI: −6.6 to 2.4); t(44) = 0.94, p = 0.35 (independent-samples t-test). Grade distributions for each scale were compared as a whole (Fisher’s exact test: KSS p = 0.95; Johner and Wruhs p = 0.84) MIPPO: minimally invasive percutaneous plate osteosynthesis; IMILN: intramedullary interlocking nailing; KSS: Knee Society Score; LEFS: Lower Extremity Functional Scale

Outcome	MIPPO (n = 24)	IMILN (n = 22)
Mean KSS (95% CI)	80.2 (75.7–84.7)	80.8 (76.1–85.5)
KSS excellent (≥ 85), n (%)	11 (45.8)	11 (50.0)
KSS good (70–84), n (%)	6 (25.0)	6 (27.3)
KSS fair (60–69), n (%)	5 (20.8)	4 (18.2)
KSS poor (< 60), n (%)	2 (8.3)	1 (4.5)
Mean LEFS (95% CI)	65.2 (62.2–68.2)	67.3 (64.1–70.5)
Johner and Wruhs excellent, n (%)	12 (50.0)	10 (45.4)
Johner and Wruhs good, n (%)	6 (25.0)	8 (36.4)
Johner and Wruhs fair, n (%)	4 (16.7)	3 (13.6)
Johner and Wruhs poor, n (%)	2 (8.3)	1 (4.6)

Weight-bearing timeline

Partial weight-bearing was initiated at a median of three days (range: two to four days) after IMILN versus a median of three weeks (range: three to four weeks) after MIPPO; this difference was significant on the Mann-Whitney U test (p < 0.001). Time to full weight-bearing, governed by radiographic callus, was similar between groups (mean 9.4 vs. 9.7 weeks; independent-samples t-test, p = 0.51).

**Figure 2:**
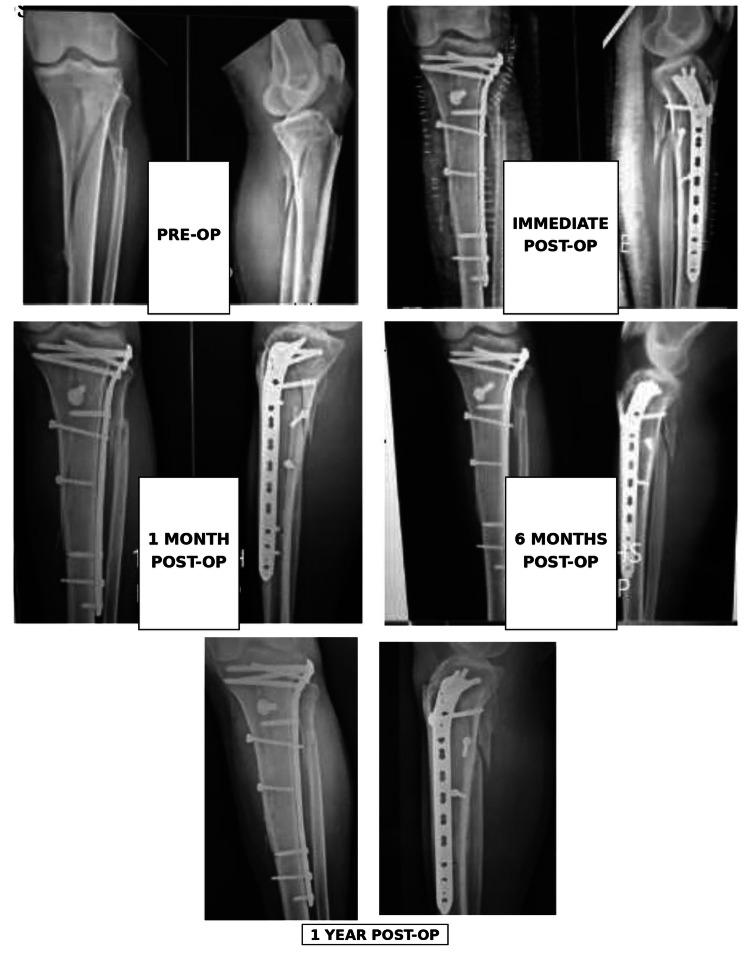
Representative MIPPO case showing radiographic progression Top row: preoperative radiographs and immediate postoperative radiographs after submuscular locking compression plate fixation. Middle row: one-month and six-month follow-up showing progressive callus formation. Bottom row: 12-month radiographs (anteroposterior and lateral) demonstrating complete radiological union with maintained axial alignment MIPPO: minimally invasive percutaneous plate osteosynthesis

**Figure 3:**
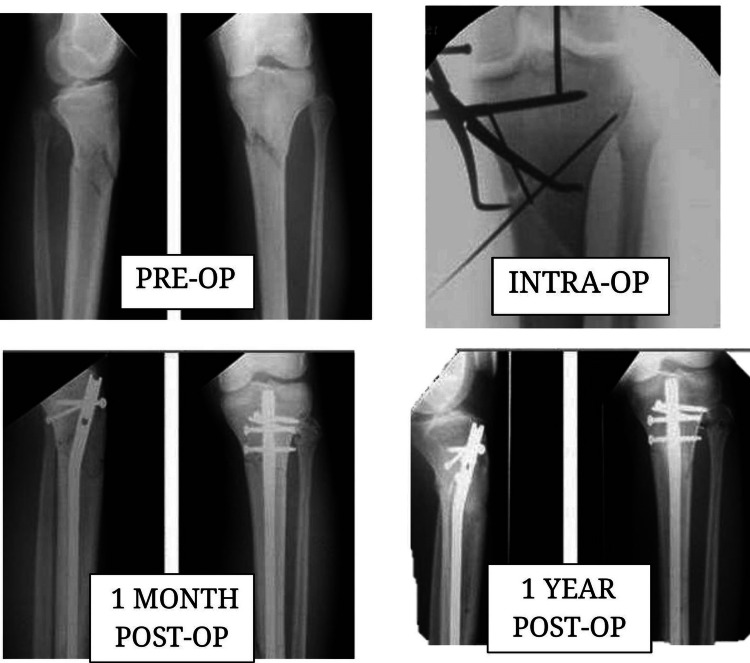
Representative IMILN case with Poller-screw adjunct Top left: preoperative anteroposterior and lateral radiographs (PRE-OP). Top right: Intraoperative fluoroscopy demonstrating Poller (blocking) screw placement to neutralize apex-anterior tendency during nail insertion; a proximal-lateral entry point was used. Bottom left: one-month postoperative radiographs after interlocking nail fixation with Poller-screw augmentation. Bottom right: 12-month follow-up radiographs demonstrating complete radiological union with maintained alignment IMILN: intramedullary interlocking nailing

**Figure 4:**
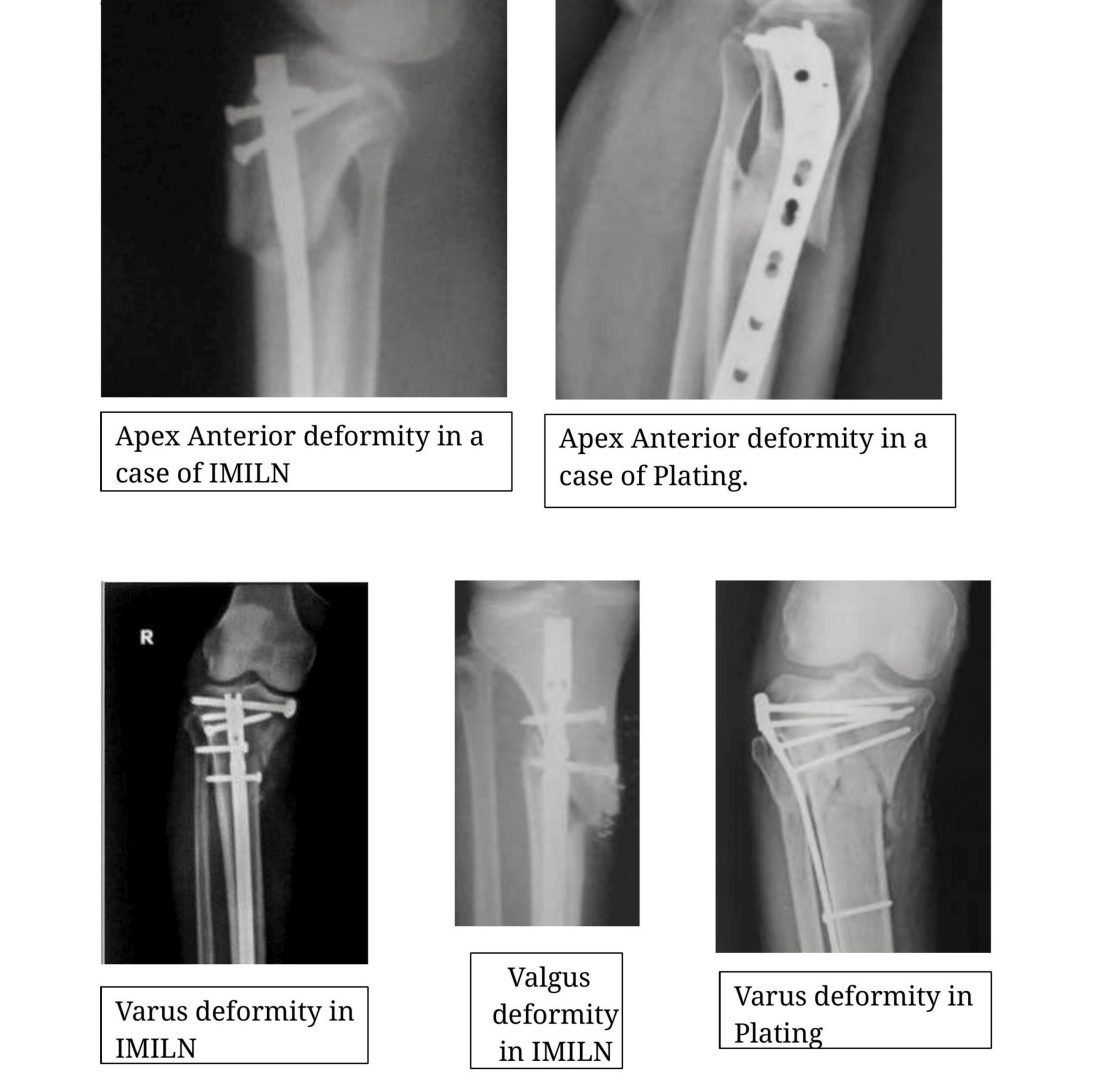
Malreduction patterns Top row: apex-anterior deformity after IMILN and after MIPPO. Bottom row: varus deformity after IMILN, valgus deformity after IMILN, and varus deformity after MIPPO. Apex-anterior angulation was the most common pattern in both groups. All illustrated cases were classified as > 5° and are included in the malreduction rates reported in Table 3 IMILN: intramedullary interlocking nailing; MIPPO: minimally invasive percutaneous plate osteosynthesis

A representative MIPPO case showing radiographic progression is presented in Figure 2. A representative intraoperative image of Poller-screw augmentation in an IMILN case is shown in Figure 3. Representative malreduction patterns are illustrated in Figure 4.

## Discussion

In this prospective cohort of AO/OTA 41A2 extra-articular proximal tibial fractures, MIPPO and IMILN produced similar union rates, malreduction rates, and one-year functional scores. The differences between the two constructs were mainly in how rehabilitation was paced and in how often the surgeon needed adjunctive measures to obtain and hold reduction on the table. These findings are consistent with the small but expanding body of recent comparative evidence regarding this fracture pattern.

A useful contemporary comparator is the prospective matched cohort study by Teimouri et al. [13], which included 29 patients treated with plating and 30 with nailing for displaced extra-articular proximal tibial fractures, using the same Johner and Wruhs outcome instrument employed in our study [22]. They reported union rates of 93% and 97%, respectively (p = 1.0), and mean union times of approximately 18 weeks for plating and 15 weeks for nailing [13]. Our union rates (95.8% for MIPPO and 95.5% for IMILN) and mean union times (16.1 and 16.9 weeks, respectively) were comparable. The modestly slower union with plating in their cohort, which was not observed in ours, may have been attributable to their fixed three-week period of strict non-weight-bearing and a higher proportion of multi-fragmentary fractures. Teimouri et al. also reported a 23% incidence of anterior knee pain after nailing, substantially higher than the 9.1% observed in our cohort; this difference may reflect variations in entry-point technique, reaming depth, and patient-reported pain assessment methodology [13].

Kapil Mani and colleagues compared MIPPO with intramedullary nailing for extra-articular distal tibial fractures (n = 100) and observed a measurable difference favoring MIPPO in postoperative malalignment (mean 5° vs. 10.22°; p = 0.001) and a lower frequency of anterior knee pain (2% vs. 10%) [12]. Although these were distal-tibial fractures, the underlying logic translates to the proximal end: when a relatively short metaphyseal segment is being reduced over a long mid-shaft, the geometry of the nail can leave residual malalignment that a plate is better placed to neutralize [12]. Our slightly higher malreduction rate in the IMILN arm (19.2% vs. 15.4% for MIPPO) is consistent with this, although the absolute difference in our cohort was small and statistically non-significant.

The 2017 systematic review by Liu and Cen [18] and the more recent comparative work by Lim et al. [16] both indicate that union, infection, and non-union rates are broadly similar between plating and nailing for proximal tibial fractures, while operative time and time to weight-bearing tend to favor nailing. Our results align with these pooled observations. The most striking residual difference, in our series as in others, is the rate of adjunct usage during nailing (50% in our IMILN arm) versus plating (11.5%). This finding may have practical implications in resource-constrained environments because adjunct use carries additional cost and theater-time implications, particularly in resource-limited settings where Poller-screw sets, expert-tibia nails, and femoral distractors are not always reliably available.

Proximal tibial malalignment following intramedullary nailing has remained one of the most extensively discussed challenges in the management of this fracture pattern for the past three decades [4,5]. Historical infrapatellar nailing series reported malalignment rates ranging from 50% to 84%, primarily because of patellar tendon pull on the short proximal fragment and the geometric mismatch between the nail's Herzog bend and the wide proximal medullary canal [4,5]. The 19.2% malalignment rate observed in our study is more representative of contemporary practice and likely reflects the routine adoption of two technical strategies in our unit: a proximal-lateral entry point aligned with the lateral intercondylar eminence and the selective use of Poller (blocking) screws [6,7] (Figure 3). The Krettek group first described the principle of Poller screws as a means of redirecting the trajectory of the nail within metaphyseal bone [7]. Subsequently, Ricci et al. reported that the judicious use of blocking screws can reduce residual malalignment to below 10% in proximal tibial nailing [6,7].

Suprapatellar (semi-extended) nailing has become an increasingly attractive option, largely because the near-extended knee position reduces tension on the patellar tendon during nail insertion, thereby minimizing the apex-anterior deforming force on the proximal fragment [5,14]. The meta-analysis by Wang et al. [14] combined studies comparing suprapatellar and infrapatellar approaches and reported shorter operative times, fewer fluoroscopy exposures, better reduction accuracy, and lower Visual Analog Scale (VAS) pain scores with the suprapatellar approach. Teixidor-Serra et al., in a multivariable analysis of 293 consecutive suprapatellar nailings, achieved acceptable alignment (≤5° in both planes) in 92.8% of cases, identifying a surgical delay of ≥7 days and age >50 years as the strongest predictors of malalignment [15].

Similarly, the recent prospective comparative study by Santhanam et al. [17] and the contemporaneous report by Lim et al. [16] suggested that the suprapatellar approach yields outcomes comparable to the traditional infrapatellar technique, while offering potential advantages in fracture alignment and postoperative knee comfort [16,17]. Because our centre did not adopt the suprapatellar technique during the study period, it remains uncertain whether the 19.2% IMILN malreduction rate observed in our cohort could have been further reduced with a semi-extended approach. This question warrants further investigation and will be explored in future studies.

In the MIPPO arm, the 15.4% malreduction rate fell within the 10-20% range reported in larger plating series [3,8]. Apex-anterior deformity was the most frequent malalignment pattern in both groups, highlighting that successful indirect reduction requires a well-positioned lateral fluoroscopic projection, careful interpretation of cortical contours, and a low threshold for repeat imaging whenever alignment appears suboptimal.

The wound infection rates observed in our series (superficial: 16.7% for MIPPO and 13.6% for IMILN; deep: 4.2% for MIPPO and 0% for IMILN) were higher than those reported in plating series limited to closed injuries but were consistent with cohorts that, like ours, included a substantial proportion of open fractures [8,12]. As 42.3% of our cases were open fractures, most superficial infections occurred in injuries classified as Gustilo-Anderson grade I or II at the time of presentation, and all resolved with antibiotics and wound care. The single deep infection occurred in a Gustilo-Anderson grade II open fracture treated with MIPPO, underscoring the well-recognized interaction between subcutaneous implants and a soft-tissue envelope already compromised at the time of injury.

From a rehabilitation perspective, the load-sharing nature of the intramedullary nail permitted partial weight-bearing within two to three days, whereas MIPPO patients commenced weight-bearing after approximately three to four weeks. In a predominantly working-age population, the short-term advantages of earlier mobilization are clinically meaningful, including shorter inpatient stays, earlier independence, and reduced disuse-related muscle atrophy; however, this did not result in measurable gains in 12-month functional scores, consistent with the broader pattern reported in the literature [8,13]. Whether earlier mobilization translates into more durable long-term benefits, such as improved bone mineral density, earlier return-to-work, or enhanced productivity, remains uncertain and requires longer follow-up and patient-reported outcome data that were not collected in this study.

Anterior knee pain occurred in 9.1% of our IMILN patients, at the lower end of the reported 7-40% range cited for infrapatellar approaches [11,14]. Several factors likely contributed, including the small cohort size, the routine use of a proximal-lateral entry point, and our standard practice of countersinking the nail only 2-3 mm below the entry cortex. Implant-related irritation in the MIPPO arm affected 8.3% of patients; none required premature hardware removal during the study period, but longer-term follow-up is likely to identify additional cases in which symptomatic hardware removal becomes desirable.

Neither technique is categorically superior on the basis of our data, and the broader literature is largely consistent with this finding [13,16,18]. Importantly, the confidence intervals around the between-group differences were narrow and centred close to zero, indicating that any true clinical difference between MIPPO and IMILN is likely to be minimal. Implant selection may be guided by three broad considerations: first, fracture morphology, where very proximal fractures with a short metaphyseal segment tend to favor locking plate fixation, while segmental fractures with a sufficient proximal segment can be effectively stabilized with a nail; second, soft-tissue status, where a compromised anterolateral envelope may favor intramedullary nailing as the safer option; and third, the locally available instrumentation, where reliable access to Poller screws, a femoral distractor, and a suprapatellar instrumentation set makes the nailing technique considerably more versatile and forgiving [6,7]. In centres without these resources, MIPPO is often the more predictably reproducible option, albeit at the cost of a more conservative rehabilitation timeline. This is particularly relevant to trauma services in much of South Asia, where instrumentation availability, operating theatre time, and follow-up logistics often influence implant choice as strongly as fracture morphology does.

Strengths of this study include its prospective design, the relatively homogeneous cohort of AO/OTA 41A2 fractures, the use of three validated outcome instruments, and a minimum follow-up of one year. Restricting inclusion to 41A2 fractures helped reduce the interpretive heterogeneity introduced by mixed 41A3, 41B, and 41C subgroups in earlier comparative series.

Limitations

This study has several important limitations. First, the non-randomized, alternating allocation design lacked allocation concealment and blinding, thereby increasing the risk of selection bias. Importantly, all clinical scores and multiplanar radiographs were assessed by the treating surgical team rather than an independent blinded panel, introducing potential observer bias. Second, the study did not include a formal a priori sample size calculation; as a result, it may be underpowered, increasing the risk of a type II error and limiting confidence in the observed equivalence between groups. Third, the use of a per-protocol rather than an intention-to-treat analysis resulted in the exclusion of patients lost to follow-up (IMILN: 15.4%, 4/26; MIPPO: 7.7%, 2/26), which may have introduced attrition bias and affected estimates of treatment effect. Fourth, restricting inclusion to homogeneous AO/OTA 41A2 fractures enhanced internal validity but reduced external validity, limiting applicability to more complex proximal tibial fracture patterns.

Fifth, regarding surgeon-related bias, although procedures were limited to a unified core team of two senior surgeons to maintain technical standardization, discretionary use of reduction adjuncts could still reflect individual operator preference. Sixth, the 12-month tracking window represents a short-term horizon that fails to document late hardware complications, long-term implant removal rates, or secondary post-traumatic arthrosis. Seventh, outcomes were restricted to region-specific metrics (KSS and LEFS) rather than holistic health-related quality-of-life tools like the Short Form 36 (SF-36) [23] or EuroQol 5-Dimension (EQ-5D) [24]. Finally, because our intramedullary arm was confined entirely to traditional infrapatellar patellar tendon-splitting entry trajectories, the performance and alignment precision of modern suprapatellar or semi-extended nailing techniques remain completely untested at this center.

Clinical relevance and future directions

For practising orthopedic trauma surgeons, the message is essentially pragmatic: when correctly executed, both MIPPO and IMILN are expected to achieve acceptable union and one-year functional outcomes in AO/OTA 41A2 fractures. Each technique has recognizable failure modes that should inform preoperative counseling and intraoperative planning-namely anterior knee pain and malalignment with nailing, and soft-tissue irritation alongside the need for a more cautious rehabilitation protocol with plating. Future priorities should include multicentre prospective trials with concealed randomization, head-to-head comparison of suprapatellar nailing versus MIPPO in this specific fracture subgroup, routine inclusion of broader patient-reported outcome measures, longer follow-up to capture late hardware-related events, and health-economic analyses relevant to resource-constrained and middle-income trauma systems.

## Conclusions

In skeletally mature adults with AO/OTA 41A2 extra-articular proximal tibial fractures, MIPPO and IMILN achieved comparable union rates (≈95.6%) and one-year functional outcomes, with no statistically significant differences between techniques, within the constraints of this small, single-centre cohort. IMILN permitted earlier mobilization but required intraoperative adjuncts more frequently and was associated with anterior knee pain. MIPPO was associated with minimal knee-related morbidity, but necessitated a more conservative weight-bearing protocol. Implant selection should be guided by fracture morphology, soft-tissue status, and available instrumentation, rather than a fixed preference for one construct over the other; however, these findings should not be extrapolated to newer semi-extended or suprapatellar nailing techniques. Larger multicentre studies with allocation concealment, broader patient-reported outcome measures, and direct comparison with suprapatellar nailing are needed to further refine these recommendations.

## References

[1] Court-Brown CM, Caesar B (2006). Epidemiology of adult fractures: a review. Injury.

[2] Nork SE, Barei DP, Schildhauer TA, Agel J, Holt SK, Schrick JL, Sangeorzan BJ (2006). Intramedullary nailing of proximal quarter tibial fractures. J Orthop Trauma.

[3] Cole PA, Zlowodzki M, Kregor PJ (2004). Treatment of proximal tibia fractures using the less invasive stabilization system: surgical experience and early clinical results in 77 fractures. J Orthop Trauma.

[4] Lang GJ, Cohen BE, Bosse MJ, Kellam JF (1995). Proximal third tibial shaft fractures. Should they be nailed?. Clin Orthop Relat Res.

[5] Tornetta P 3rd, Collins E (1996). Semiextended position of intramedullary nailing of the proximal tibia. Clin Orthop Relat Res.

[6] Ricci WM, O'Boyle M, Borrelli J, Bellabarba C, Sanders R (2001). Fractures of the proximal third of the tibial shaft treated with intramedullary nails and blocking screws. J Orthop Trauma.

[7] Krettek C, Stephan C, Schandelmaier P, Richter M, Pape HC, Miclau T (1999). The use of Poller screws as blocking screws in stabilising tibial fractures treated with small diameter intramedullary nails. J Bone Joint Surg Br.

[8] Lindvall E, Sanders R, Dipasquale T, Herscovici D, Haidukewych G, Sagi C (2009). Intramedullary nailing versus percutaneous locked plating of extra-articular proximal tibial fractures: comparison of 56 cases. J Orthop Trauma.

[9] Bhandari M, Audige L, Ellis T, Hanson B (2003). Operative treatment of extra-articular proximal tibial fractures. J Orthop Trauma.

[10] Avilucea FR, Triantafillou K, Whiting PS, Perez EA, Mir HR (2016). Suprapatellar intramedullary nail technique lowers rate of malalignment of distal tibia fractures. J Orthop Trauma.

[11] Chan DS, Serrano-Riera R, Griffing R (2016). Suprapatellar versus infrapatellar tibial nail insertion: a prospective randomized control pilot study. J Orthop Trauma.

[12] Kapil Mani KC, Pangeni BR, Marahatta SB, Sigdel A, Amuda KC (2022). Comparative study between intramedullary interlocking nailing and minimally invasive percutaneous plate osteosynthesis for distal tibia extra-articular fractures. Chin J Traumatol.

[13] Teimouri M, Mirghaderi P, Parry JA, Ziaei A, Salimi M, Tahririan MA (2023). Intramedullary nail versus minimally invasive plate osteosynthesis for displaced extraarticular proximal tibia fractures: a prospective comparative cohort study. Eur J Orthop Surg Traumatol.

[14] Wang Z, Xiong X, Lu Z, Gao Y (2024). A systematic review and meta-analysis comparing suprapatellar versus infrapatellar approach intramedullary nailing for tibal shaft fractures. Eur J Trauma Emerg Surg.

[15] Teixidor-Serra J, Andrés-Peiró JV, García-Sanchez Y (2024). Outcomes and their predictors in suprapatellar nailing for tibia fractures. Multivariable analysis of 293 consecutive cases. Eur J Trauma Emerg Surg.

[16] Lim S, Song HK, Kim TH, Park DY, Lee JW, Chung JY (2024). Suprapatellar intramedullary nail combined with screw fixation has comparable surgical outcomes to minimally invasive locking plate fixation in ipsilateral tibial plateau and shaft fractures. Arch Orthop Trauma Surg.

[17] Santhanam SS, Velayutham S, Krishnan P, Albert A, Ramanujam B (2024). Comparison of efficacy of suprapatellar and infrapatellar approaches for intramedullary interlocking nailing of tibia in patients with tibial fracture. Cureus.

[18] Liu X, Cen S (2017). Comparison of intramedullary nailing and plate fixation for the management of extra-articular proximal tibial fractures: a systematic review and meta-analysis. J Orthop Surg Res.

[19] Gustilo RB, Anderson JT (1976). Prevention of infection in the treatment of one thousand and twenty-five open fractures of long bones: retrospective and prospective analyses. J Bone Joint Surg Am.

[20] Insall JN, Dorr LD, Scott RD, Scott WN (1989). Rationale of the Knee Society clinical rating system. Clin Orthop Relat Res.

[21] Binkley JM, Stratford PW, Lott SA, Riddle DL (1999). The Lower Extremity Functional Scale (LEFS): scale development, measurement properties, and clinical application. Phys Ther.

[22] Johner R, Wruhs O (1983). Classification of tibial shaft fractures and correlation with results after rigid internal fixation. Clin Orthop Relat Res.

[23] Ware JE Jr, Sherbourne CD (1992). The MOS 36-item short-form health survey (SF-36). I. Conceptual framework and item selection. Med Care.

[24] EuroQol Group (1990). EuroQol--a new facility for the measurement of health-related quality of life. Health Policy.

